# Highly selective cleavage C–O ether bond of lignin model compounds over Ni/CaO–H-ZSM-5 in ethanol

**DOI:** 10.1186/s13065-019-0557-z

**Published:** 2019-03-26

**Authors:** J. Guo, Yu L. Ma, Jia Y. Yu, Yu J. Gao, Ning X. Ma, Xiao Y. Wu

**Affiliations:** State Key Laboratory of High-efficiency Coal Utilization and Green Chemical Engineering, College of Chemistry and Chemical Engineering, Ningxia University, Helanshan Rd. 539, Yinchuan, 750021 China

**Keywords:** Lignin model compound, Ni, CaO, H-ZSM-5, Hydrogenolysis

## Abstract

Herein, 2-(2-methoxyphenoxy)-1-phenylethanol (β-*O*-4), 2-methoxyphenyl anisole (α-*O*-4) and 4-phenoxyphenol (4-*O*-5) were selected as typical lignin model compounds. Given the effectiveness of traditional acid–base catalysts for lignin depolymerisation, a novel Ni/CaO–H-ZSM-5(60) catalyst was prepared to investigate the difficulty level of C–O bond of three model compounds cleavage in ethanol. It was observed that Ni/CaO–H-ZSM-5(60) had prominent performance on the C–O bond cleavage at very mild conditions (140 °C, 1 MPa H_2_). Among them, the C–O bond of α-*O*-4 and β-*O*-4 could be completely cleaved within 60 min. Although the C–O bond of 4-*O*-5 had high bond energy, 41.2% of conversion was occurred in 60 min. The introduction of CaO could regulate the acidity of H-ZSM-5 to enhance the ability to break C–O bonds. Moreover, the possible pathways of C–O ether bonds in three lignin model compounds cleavage were proposed in order to selectively obtain target products from the raw lignin degradation.

**Electronic supplementary material:**

The online version of this article (10.1186/s13065-019-0557-z) contains supplementary material, which is available to authorized users.

## Introduction

As one of the most abundant renewable energy sources today, lignocellulosic biomass, which can generate zero-carbon fuels and green fine chemical, has the advantages of widely distributed, renewable and clean [1]. Hemicellulose (20–35%), cellulose (35–50%), and lignin (10–25%) are mainly three components in lignocellulosic biomass. Among them, extensive efforts have been devoted to convert hemicellulose and cellulose into fine chemicals and fuels for a long time [2–4].

**Fig. 1:**
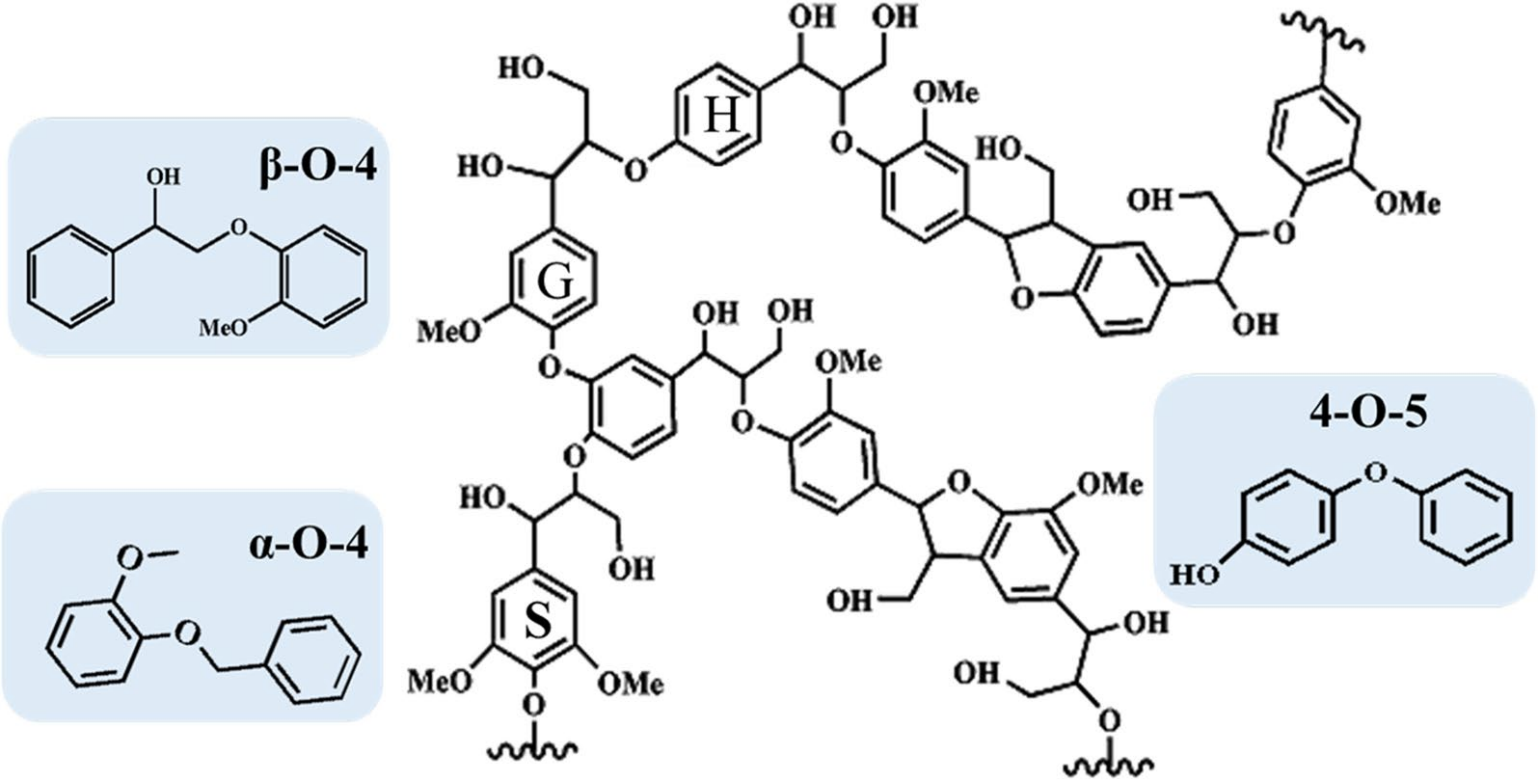
Representative structure of native lignin and three lignin model compounds for the β-*O*-4, α-*O*-4, and 4-*O*-5 linkages

In contrast, lignin, as one of the main components of lignocellulosic biomass, is a three-dimensional amorphous polymer combining with sinapyl alcohol (S), coniferyl alcohol (G) and p-coumaryl alcohol (H) (Fig. 1) [5, 6]. In addition, lignin has sufficient mechanical strength and hardness, and these stable properties make it be difficult to depolymerization. Therefore, researches on the application of lignin were still in the primary stage and some industries even treat it as a waste product [7]. However, lignin with unique structure, chemical properties and high energy density was known as the only renewable aromatic compound in nature, and a great deal of fine chemicals and fuels could be obtained from it, especially aromatics [8]. At present, it has been confirmed that the methods of pyrolysis [9], noble metal catalysis [10], acid catalysis [11] and oxidation [12] have a prominent effect on the degradation of lignin. However, most of these methods have quite a few drawbacks. For example, pyrolysis requires either high temperature (> 250 °C) or high pressure (> 4 MPa), and the products would re-polymerize at high temperatures during the reaction. Noble metal catalysts could achieve high conversion of biomass in relatively short time, but its high cost and low availability limit their applications in large scale processes [13]. Application of acid catalysts could lead to equipment corrosion, which was not conducive to recovery, and even requires high temperatures (> 340 °C) to obtain aromatics and gases [14, 15]. The methods of oxidation lignin usually caused irreparable damage to aromatic structure in lignin and the lignin would be deeply oxidized to COx and H_2_O, reducing the yield of lignin products [16, 17]. Amongst all the methods we known, acid–base catalytic degradation of lignin has been widely studied. Konnerth et al. [18] proved that strong bases such as NaOH could break the C–O bonds of dimeric lignin model compounds in aqueous solutions with the high selectivity. Bengoechea et al. [19] found that the stable Lewis acidity in the alumina support was beneficial to the conversion of lignin, and proposed suitable acidic conditions were indispensable for efficient depolymerization of lignin. With the sustainable development of green chemistry, the solid acid–base catalysts have attracted much attention due to its low corrosivity and high catalytic selectivity, especially hindering the formation of tar by-products. Wherein, metal oxides were considered as outstanding catalysts for lignin conversion, and some researches were dedicated on them [20, 21]. However, some metal oxides such as MgO and CaO as catalysts also have many drawbacks such as small surface area and easy aggregation during the reaction. In order to improve the catalytic activities of metal oxides, a large number of materials has been found to be suitable as catalytic supports for the lignin conversion and have been widely studied. The materials of hydrotalcite (HTC) [22, 23], activated carbon [24], alumina [25], silica [26], and zirconia [27] have also been reported to be effective remarkably in cleavage of C–O bonds in lignin model compounds. Compared with them, ZSM-5 zeolites with unique shape are suitable for aromatization and cracking reactions due to the properties of high temperature resistance, ideal pore size and suitable acidity. However, although the acidic sites of ZSM-5 were benefit for the hydrodeoxygenation reaction, the hydrocarbon conversion process easily forms coke, resulting in loss of catalytic activity and complete inactivation. Therefore, in order to improve the activity of catalyst, it was particularly important for the modification of ZSM-5. Zhang et al. [28] found that the Fe-modified ZSM-5 zeolite could significantly increase the yield of olefins. Similarly, Niu et al. [29] studied the catalytic performance of Zn-modified ZSM-5 zeolite in the conversion of methanol to aromatic hydrocarbons (MTA) and found that small crystal of Zn/H-ZSM-5 can greatly improve aromatics selectivity and catalyst. In addition, metal nickel, which has similar electronic properties with palladium or platinum, was extensively used to modify HZSM-5 to improve the activity of hydrodeoxygenation of the catalysts [30, 31].

In this study, 2-(2-methoxyphenoxy)-1-phenylethanol, 2-methoxyphenyl anisole, 4-phenoxyphenol were used as lignin model compounds for the β-*O*-4, α-*O*-4 and 4-*O*-5 bonds, respectively. We selected H-ZSM-5 zeolite with Si/Al_2_ ratio of 60, and modified it by incorporating the metal Ni (Ni loading = 45 wt%) and CaO with the deposition–precipitation (DP) method to prepare Ni/CaO–H-ZSM-5(60) catalyst. The efficiency of the cleavage of C–O bond in three typical lignin model compounds was investigated. Moreover, according to the results, the possible pathways of C–O bonds cleavage of lignin model compounds were proposed, respectively.

## Materials and methods

### Materials

Chemicals and reagents were received from commercial suppliers: Ni (NO_3_)_2_·6H_2_O (Tianjin Kai-Chemical Reagent Co. Ltd), Ca (NO_3_)_2_·4H_2_O (Aladdin), NaOH (Sinopharm Chemical Reagent Co. Ltd), Na_2_CO_3_ (Sinopharm Chemical Reagent Co. Ltd), 4-*O*-5 lignin model compound (Aladdin), β-*O*-4 lignin model compound and α-*O*-4 lignin model compound were prepared in our laboratory, as seen below.

H-ZSM-5 zeolite with Si/Al_2_ ratio of 60 from Nan Jin Huang Ma was used for the preparation of the catalyst involved in the experiment. In addition, H-ZSM-5(60) zeolite was used after calcination at 550 °C for 6 h and all chemicals were of analytical grade and used without any purification.

### Preparation of lignin model compounds

2-(2-Methoxyphenoxy)-1-phenylethanol (β-*O*-4) was synthesized in a two-step process [32]. 2-Bromoacetophenone (50 mmol, 9.9 g), guaiacol (50 mmol, 6.2 g) and K_2_CO_3_ (137 mmol, 19 g) were added to a 100 mL flask. Stirred it for 5 min after adding 50 mL of acetonitrile, then, added KI (1.2 mmol, 0.2 g) and stirred it continued for 10 min. Next, the reaction mixture was stirred at reflux temperature for 24 h before recrystallized from ethanol. Then, the obtained compound was dissolved in the mixture of THF:H_2_O (5:1) (60 mL), and sodium borohydride (37 mmol, 1.4 g) was added batch-wise, next, the mixture was stirred at room temperature overnight. After the reaction, the mixture was diluted with dilute hydrochloric acid until no bubbles were formed. Later, 80 mL of ethyl acetate was added thereto, and the organic phase was washed three times with 30 mL of saturated brine. β-*O*-4 model compound obtained after solvent evaporation (shown in Additional file 1: Figure S1).

The preparation processes of 2-methoxyphenyl anisole (α-*O*-4) and 1-methoxy-2-phenylethoxybenzene were the same as that of β-*O*-4, and only the reactant materials were different. 2-methoxyphenyl anisole (α-*O*-4) was prepared from 2-bromoethylbenzene (50 mmol, 9.2 g) and guaiacol (50 mmol, 6.2 g). Preparation of 1-methoxy-2-phenylethoxybenzene from benzyl bromide (50 mmol, 8.6 g) and guaiacol (50 mmol, 6.2 g). In addition, the structure and purity of the model compounds prepared were confirmed by the H^1^NMR spectrum (shown in Additional file 1: Figure S1).

### Catalyst synthesis

The nickel and calcium oxide nanoparticles were introduced into the zeolites using deposition–precipitation (DP) method, a typical synthetic method, and the specific procedure was carried out as follows [33].

0.30 g of Ca (NO_3_)_2_·4H_2_O (Ca loading = 5 wt%) and 2.23 g of Ni (NO_3_)_2_·6H_2_O (Ni loading = 45 wt%) were dissolved in deionized water, denoted as solution A. The mixture of NaOH and Na_2_CO_3_ was prepared at a concentration of 0.25 mol/L and 0.8 mol/L, respectively, called solution B. In another 300 mL beaker, 0.48 g of H-ZSM-5(60) and 50 mL of deionized water were introduced under fast stirring at 60 °C (solution C). In the next step, solution B was added dropwise to the beaker C, until the pH of the solution in the beaker C was maintained between 10 and 11. Then, solution A was added slowly to the beaker C under vigorous stirring and light green precipitation was observed. Throughout the precipitation process, the pH of the solution in the beaker C was kept it around 10 in order to precipitation of all metal cations. After that, the substance was stirred at 60 °C for 16 h before dried overnight at 80 °C and calcined in a flow of air at 400 °C for 5 h (heat rate 1 °C/min). Next, the freshly synthesized material was reduced in a flow of H_2_ at 570 °C for 2.5 h (flow rate: 40–45 mL/min) and passivated in a flowing 5% O_2_/N_2_ (flow rate: 7–8 mL/min) for 12 h at room temperature. The catalysts were prepared by a specific procedure shown in Additional file 2: Figure S2. In order to explore the physicochemical properties of Ni/CaO–H-ZSM-5(60), we prepared CaO–H-ZSM-5(60) and Ni/H-ZSM-5(60) catalyst at the same way.

### Analytical methods

#### Characterization of catalyst

Prior to the reactions, detailed characterizations of the fresh or used catalyst were carried out. X-Ray diffraction (XRD). The powder XRD patterns were recorded on a Dmax2200PC (Rigaku) diffractometer with a Cu Kα1 radiation source (λ = 0.1540 nm), operating at 40 kV and 40 mA. Transmission electron microscopy (TEM). The TEM images were taken with a JEOL model JEM 2010 EX microscope instrument, and the accelerating voltage was 200 kV. Brunauer–Emmett–Teller (BET). The N_2_ adsorption–desorption experiments were performed at 77 K using Micromeritics ASAP2010 surface area Analyzer. The specific surface area, pore volume and pore size distribution were obtained using BET, N_2_ adsorption–desorption isotherms and Barrett–Joyner–Halenda (BJH) methods. Fourier transform infrared spectroscopy (FT-IR). The acid sites in supports were determined from FT-IR on a Thermo Nicolet 380. The sample was pretreated in a vacuum at 500 °C for 1 h before adsorption of pyridine for 30 min at room temperature, and then desorbed at 150 °C, 250 °C and 350 °C for 30 min. Determination of leaching content of Ca and Ni by inductively coupled plasma atomic emission spectrometry (ICP-AES).

#### Catalytic tests and product analysis

In a typical reaction, 0.61 g β-*O*-4 lignin model compound (2.5 mmol) and 0.102 g active catalyst were added to 30 mL ethanol, and loaded into a 50 mL stainless steel batch reactor. Then, the autoclave was flushed with H_2_ three times before filled 1 MPa H_2_ and the reaction were conducted at 100–250 °C with the stirring speed of 700 rpm (detailed steps as shown in Additional file 3: Figure S3). After the reaction, the reactor was placed in ice water and cooled to room temperature, then, the liquid products in the reactor were collected for further analysis.

The reaction products were qualitatively analysis by GC–MS (Agilent 19091S-433, HP-5 ms, 30 m × 250 μm × 0.25 μm), and the temperature setting program was as follows: the temperature of the injector and the detector were 260 °C and 270 °C, respectively. The initial temperature of the GC column oven was 50 °C kept for 5 min. Then the temperature was increased to 100 °C at the rate of 10 °C/min and retained for 5 min, followed by an increase to 300 °C (30 °C/min) kept for 4 min. Quantitatively analysis using GC-FID (GC-2014C, Wondacap-5, 30 m × 250 μm × 0.25 μm) without further dilution. The temperature setting program was as follows: the initial temperature of the GC column oven was 60 °C kept for 1 min, then the temperature was increased to 100 °C at the rate of 2 °C/min and retained for 1 min, followed by an increase to 260 °C (10 °C/min) kept for 2 min (Additional file 3: Figure S3). In addition, calibration the concentration of the products using the external standard, and the products of 2-methoxyphenyl anisole and 4-phenoxyphenol conversion were conducted as the same way. The conversion of model compound, as well as the yield and the selectivity of the products were calculated based on the following equation, respectively:

1 $$Conversion\left( \% \right) \, = {\text{ n}}_{\text{a}} /{\text{n}}_{\text{b}} * 100\%$$

2 $$Molar \, yield \, of \, product \, i\left( \% \right) \, = {\text{ n}}_{i} /{\text{n}}_{\text{b}} * 100\%$$

3 $$Selectivity \, of \, product\;i\left( \% \right) \, = {\text{ n}}_{i} /{\text{n}}_{\text{c}} * 100\%$$

where n_a_ and n_b_ were the moles of the model compound consumed and the model compound initially added, respectively. n_*i*_ and n_c_ were deemed as the moles of product *i* and the total product, respectively. In the above calculation, 1 mol of reactant could produce 2 mol of monomer products.

## Result and discussion

### Characterization of the catalysts

**Fig. 2:**
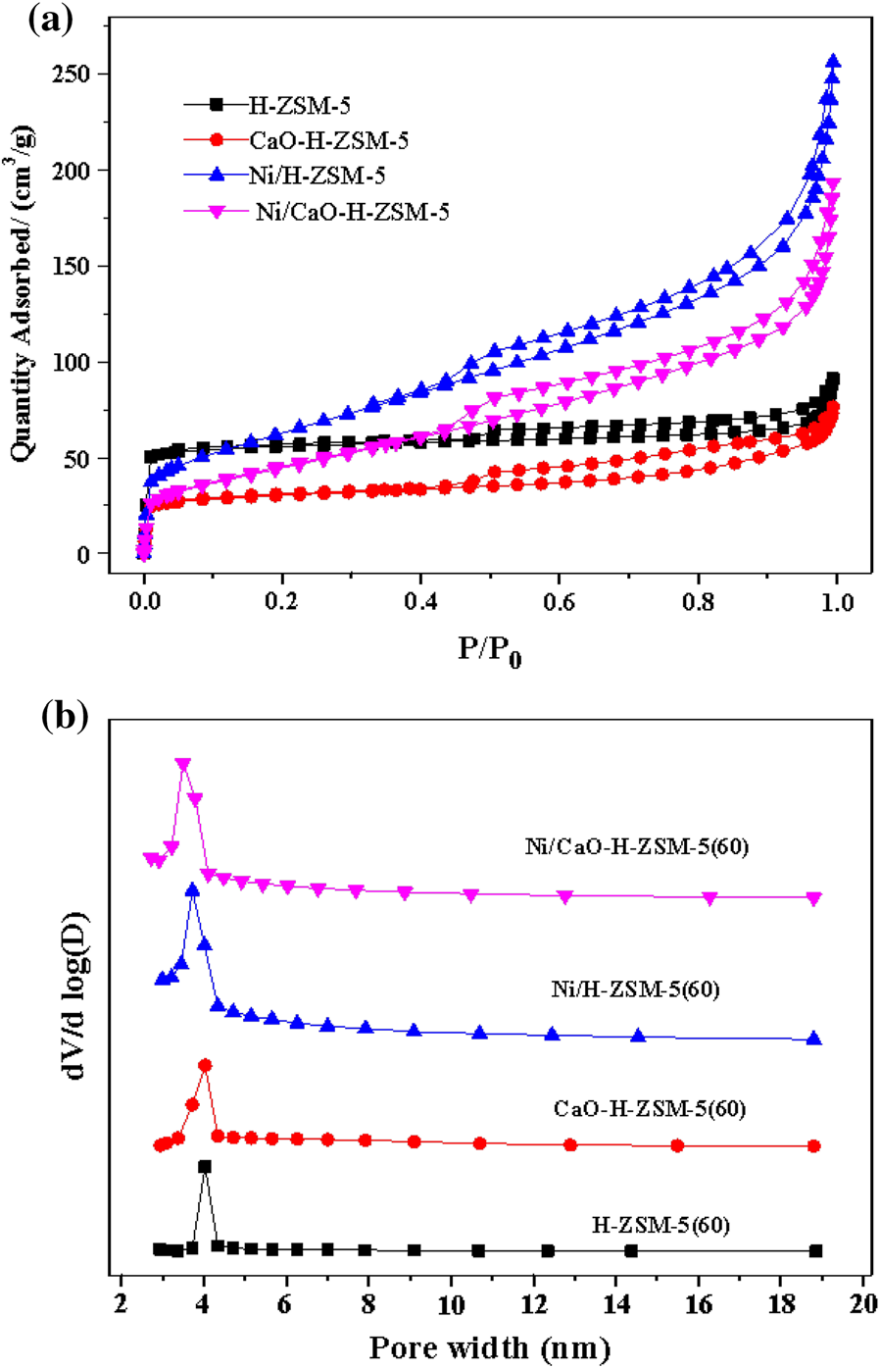
**a** N_2_ adsorption–desorption isotherms and **b** size distributions of original and modified H-ZSM-5(60) catalysts

The N_2_ adsorption–desorption isotherms and pore size distributions of all catalysts were shown in Fig. 2a, b, it could be seen that the pore size distributions of the four samples were almost identical. In addition, the Si/Al_2_ ratios of these four samples were determined by wet chemical analysis (Table 1). Compared to the original H-ZSM-5(60), the Si/Al_2_ ratios of the modified H-ZSM-5(60) catalysts were slight variation. Infrared adsorption experiments were performed using a basic probe molecule (pyridine) to determine the type of acid sites (Lewis or Brönsted). Compared with the H-ZSM-5(60), Ni/H-ZSM-5(60) has an extremely low concentration of Brönsted acid sites, but the concentration of Lewis acid sites was slight variation (Table 1), indicating that the addition of nickel would lead to the reduction of Brönsted acid sites. Some studies on the effect of nickel addition on H-ZSM-5(60) had reported that the Brönsted acid sites of the catalyst would decrease with increasing nickel concentration [34, 35], which were consist with the result we found. In addition, from the results in Table 1, finding that the addition of CaO significantly reduced the Lewis acid sites of the catalyst (Ni/CaO–H-ZSM-5(60)).

**Table 1:** The physicochemical properties of H-ZSM-5 and Ni catalysts

Catalyst	Content (wt%)		Si/Al_2_ (mol/mol)	FT-IR (μmol/g)		
	Si	Al		B	L	B/L
H-ZSM-5(60)	42.35	1.52	53.6	425.7	325.5	1.31
CaO–H-ZSM-5(60)	37.46	1.42	50.7	373.6	303.2	1.23
Ni/H–ZSM -5(60)	22.47	0.88	49.1	42.8	268.8	0.16
Ni/CaO–H-ZSM-5(60)	20.55	0.85	46.5	31.8	157.6	0.2

**Fig. 3:**
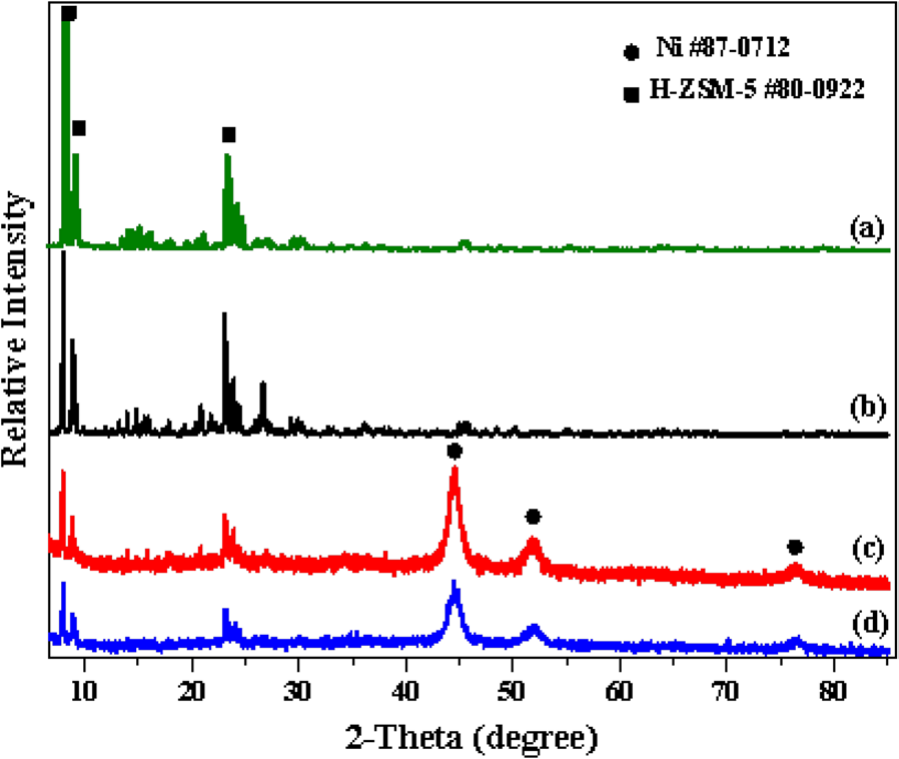
XRD pattern of different catalysts. (a) H-ZSM-5(60), (b) CaO–H-ZSM-5(60), (c) Ni/CaO–H-ZSM-5(60) and (d) Ni/H-ZSM-5(60)

**Fig. 4:**
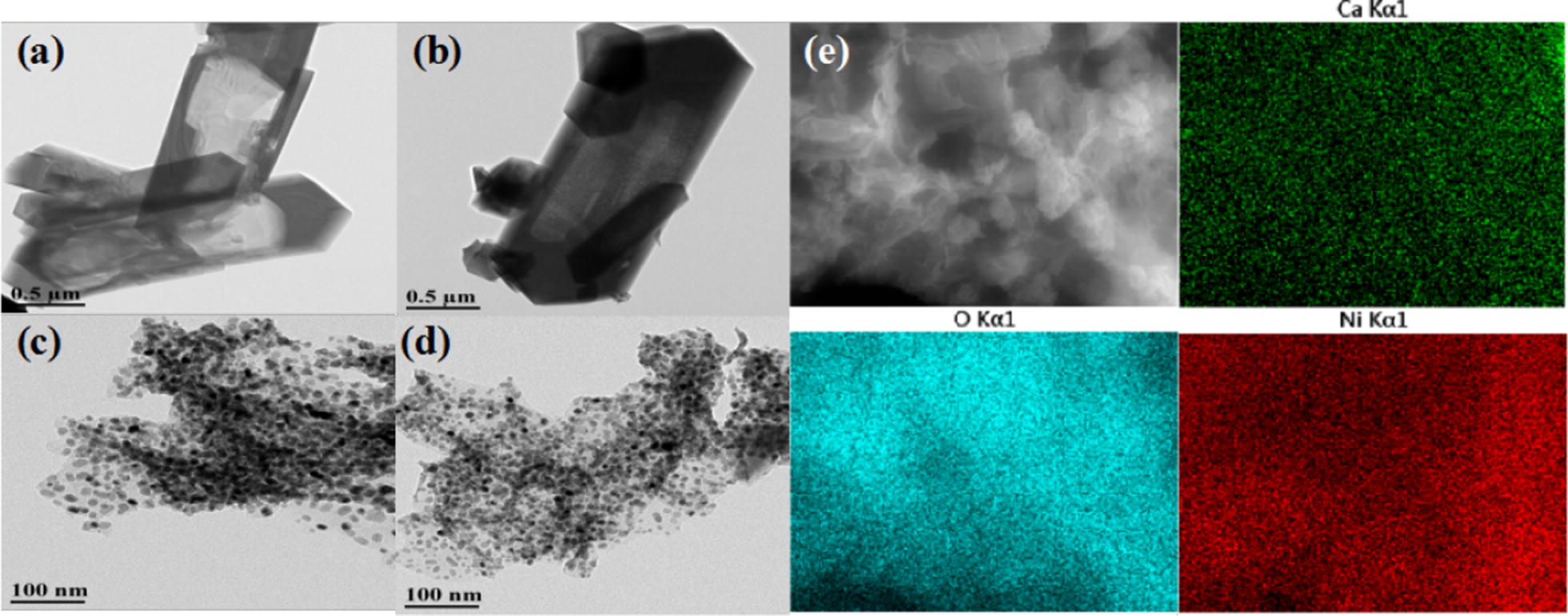
TEM images and EDX mapping of different catalysts. TEM images of **a** H-ZSM-5(60), **b** CaO–H-ZSM-5(60), **c** Ni–H-ZSM-5(60) and **d** Ni/CaO–H-ZSM-5(60). **e** EDX mapping of Ni/CaO–H-ZSM-5(60)

The powder X-ray diffraction patterns of pure H-ZSM-5(60) zeolite and three typical catalysts [CaO–H-ZSM-5(60), Ni/H-ZSM-5(60) and Ni/CaO–H-ZSM-5(60)] was presented in Fig. 3. The characteristic diffraction peaks of H-ZSM-5 phase (JCPDS#80-0922) still exist in the catalysts containing Ni and CaO, illustrating that the structure of the H-ZSM-5 was quite stable, and the addition of Ni and CaO did not change the phase structure of the H-ZSM-5, but the peak intensity had a significant decrease compared with that of the pure H-ZSM-5. The main reason was that the addition of metal (Ni) would dilute of the H-ZSM-5. There were another two possible reasons about it. One reason was that the process of catalysts preparation would affect the structure of H-ZSM-5 and result in the decrease of peak intensity of H-ZSM-5, and the higher the alkali concentration, the lower the peak intensity [36]. Another reason may be that the interaction between H-ZSM-5 and the active components (Ca, Ni) resulted in a disordered crystal structure of H-ZSM-5, which caused a decrease in the intensity of its characteristic diffraction peaks [37]. In addition, the characteristic diffraction peaks of Ni phase (JCPDS#87-0172) could found clearly, while the XRD pattern of Ni/CaO–H-ZSM-5(60) did not show any diffraction peak of CaO. Combined with the results of Fig. 4e (EDX-Mapping), it could be stated that CaO was dispersed on the surface of the catalyst.

**Fig. 5:**
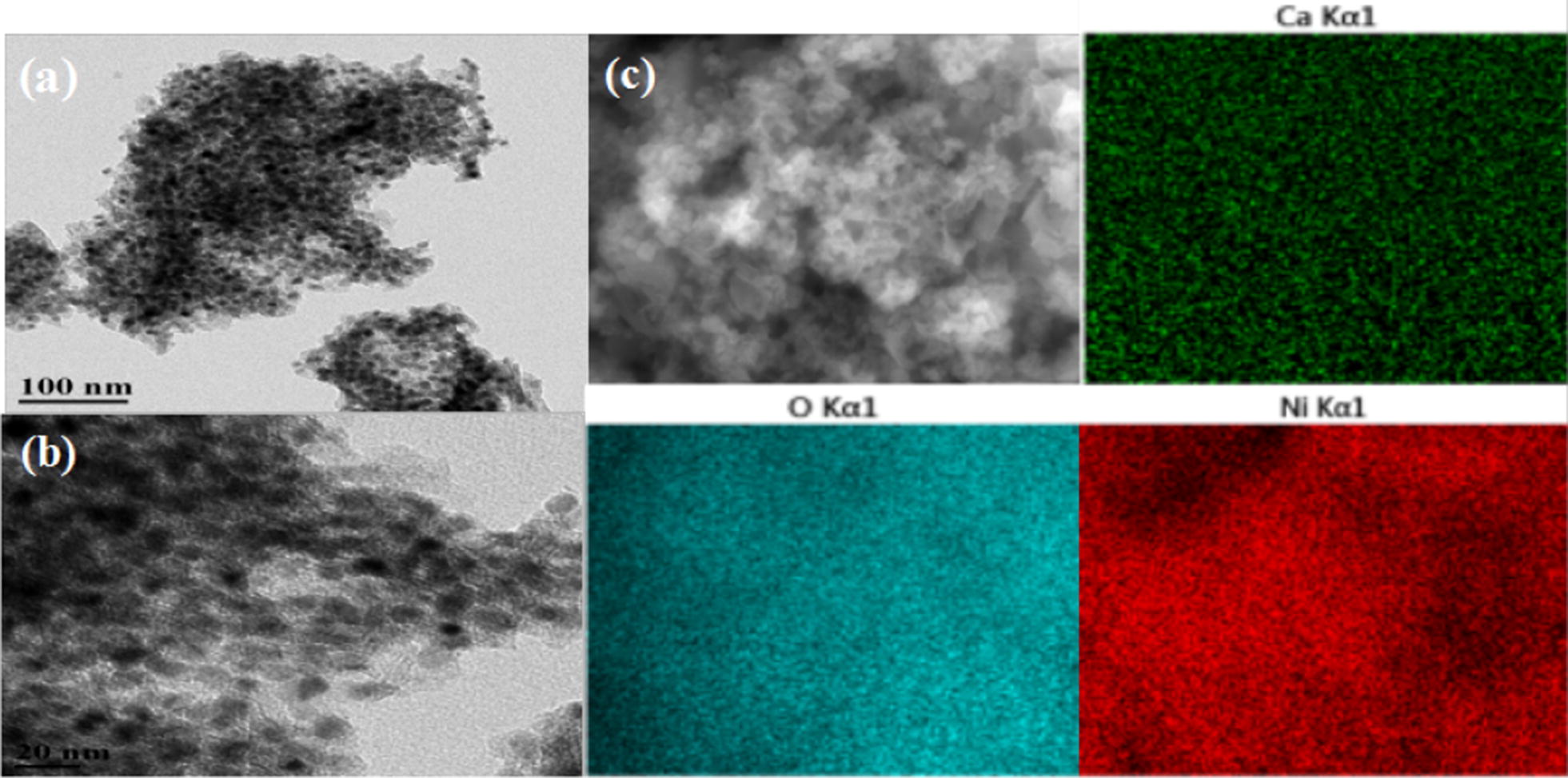
TEM images (**a**, **b**) and EDX mapping (**c**) of the used Ni/CaO–H-ZSM-5(60) catalyst

**Fig. 6:**
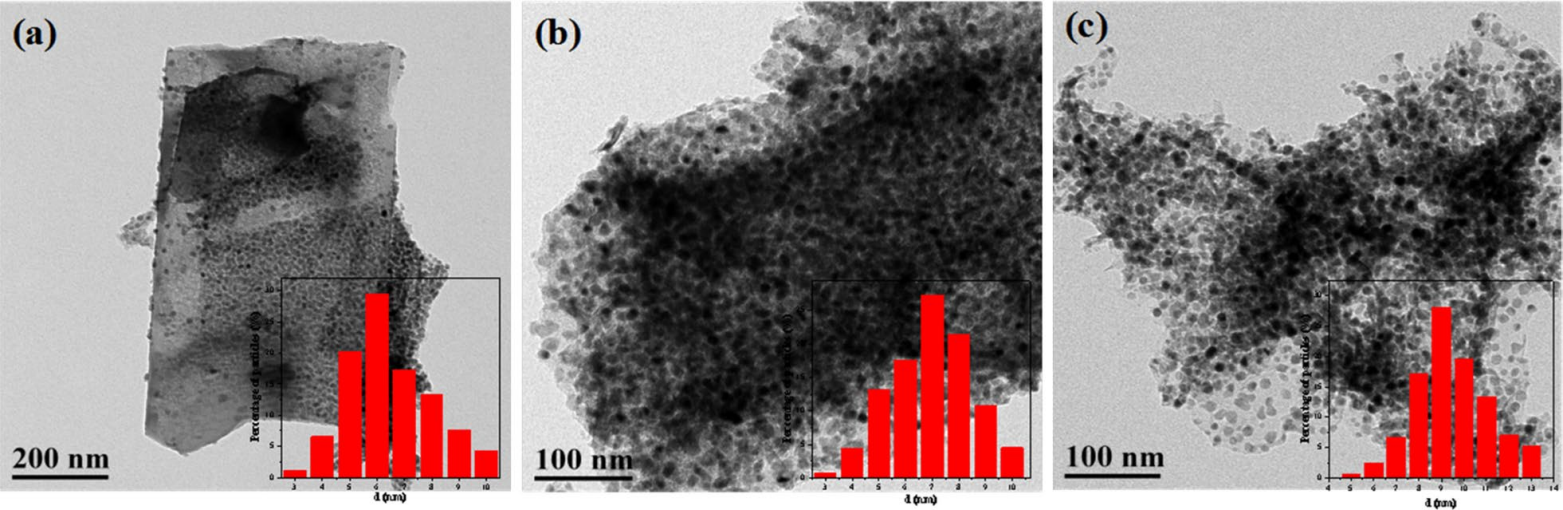
TEM images and metal particle size distributions of Ni/CaO–H-ZSM-5(60). **a** Before reaction, **b** after one cycle and **c** after three cycles

The metal dispersions of the catalysts [H-ZSM-5(60), CaO–H-ZSM-5(60), Ni/H-ZSM-5(60) and Ni/CaO–H-ZSM-5(60)] were characterized by TEM and EDX mapping, as shown in Fig. 4. It could be seen that the elements of Ni, Ca and O in Ni/CaO–H-ZSM-5(60), prepared by the deposition–precipitation method, were uniformly and regularly dispersed on the H-ZSM-5(60) zeolite (Fig. 4e). In addition, Ni nanoparticles had small particle size and uniform distribution without aggregation (Fig. 4b–d), the reason for this result was that the large specific surface area of the H-ZSM-5 zeolite was conducive to the dispersion of nickel. The used catalyst (Ni/CaO–H-ZSM-5(60)) was also characterized by TEM and EDX mapping as shown in the Fig. 5. The elements of Ni, Ca, and O were still distributed evenly on the support of H-ZSM-5 zeolite after the reaction. Additionally, the average particle size of the Ni/CaO–H-ZSM-5(60) catalyst was calculated by TEM analysis (Fig. 6). The average particle size of the Ni/CaO–H-ZSM-5(60) after the first cycle was 6.95 nm, which was similar with the particle size of the fresh Ni/CaO–H-ZSM-5(60) (6.47 nm). The result indicated that the catalyst was quite stable during the reaction. When the catalyst was cycled three times, the average particle size of Ni/CaO–H-ZSM-5(60) reached 9.47 nm, indicating that the particles of nickel had underwent significant agglomeration during the catalytic reaction.

The stability of the catalyst was determined by measuring the concentration of the metal in the liquid phase product using ICP-AES. In the first cycle, the concentrations of Ca and Ni in the liquid phase were 0.13 and 0.08 mg L^−1^, respectively. After the second cycle, the concentrations of Ca and Ni were 0.12 and 0.04 mg L^−1^, respectively. It experienced the last cycle, the concentrations of Ca and Ni were 0.18 and 0.12 mg L^−1^, respectively. The leaching concentrations of Ca and Ni were extremely low in three cycles, it explained why the Ni/CaO–H-ZSM-5(60) catalyst was still active for three cycles.

### Catalyst screening

2-(2-Methoxyphenoxy)-1-phenylethanol containing alkyl-aryl-ether linkages, was used as the reactant for testing the activity of catalyst, because it was a kind of representative β-*O*-4 lignin model compound and was most abundant in native lignin. Ethanol was selected as reaction solvent for the lignin model compounds conversion. Because the alcohol molecule could be used as a nucleophilic reagent in the C–O cracking process [38, 39], and the products dissolved in ethanol could stably maintained without separation and condensation even at a harsh condition. However, subsequent experiments had found that H_2_ provided hydrogen source during the reaction, which was essential for the cleavage of C–O ether bond.

As shown in Table 2, the conversion of β-*O*-4 model compound over H-ZSM-5(60) and CaO–H-ZSM-5(60) was extremely low, with only 2.1% and 5.3% conversion, respectively. However, over Ni/H-ZSM-5(60) and Ni/CaO–H-ZSM-5(60), the conversion of β-*O*-4 model compound reached more than 80%. The results indicated that Ni was indispensable in the hydrogenation reaction. In addition, it could be found that the addition of CaO in Ni/H-ZSM-5(60) significantly affected the product distributions of the β-*O*-4 model compound (Table 2). Over Ni/H-ZSM-5(60), about 83% of the β-*O*-4 had been converted, producing 14.8% selectivity of ethylbenzene, 11.7% selectivity of guaiacol and 73.4% selectivity of dehydration product (1-methoxy-2-phenylethoxybenzene). In contrast, over Ni/CaO–H-ZSM-5(60), about 90% of the β-*O*-4 had been converted, producing 7.4% selectivity of ethylbenzene, 42.7% selectivity of 1-phenylethanol, 49.6% selectivity of guaiacol and only 0.3% selectivity of dehydration product. In addition, 1-phenylethanol, as a reactant, was converted by these two catalysts and the results shown in Table 2. According the results, inferring that the Ni/H-ZSM-5(60) catalyst had better performance on hydrodeoxygenation, while Ni/CaO–H-ZSM-5(60) had more effective on the cleavage of ether bonds.

**Table 2:** The main product distributions of the conversion of β-*O*-4, 4-*O*-5, α-*O*-4 and 1-phenyl ethanol over different catalyst

Reactant	Catalyst	Conv. (%)	Selectivity (%)			
			Ethylbenzene	1-Phenyl ethanol	Guaiacol	1-Methoxy-2-phenethoxybenzene
β-*O*-4 model compound	H-ZSM-5(60)	2.1	–	–	47.2	10.7
	CaO–H-ZSM-5(60)	5.3	–	37.5	62.5	–
	Ni–H-ZSM-5(60)	82.8	14.8	–	11.7	73.4
	Ni–CaO–H-ZSM-5(60)	88.2	7.4	42.7	49.6	0.3
1-Phenyl ethanol	Ni–H-ZSM-5(60)	56.6	100	–	–	–
	Ni–CaO–H-ZSM-5(60)	44.0	99.2	–	–	–

Reactant	Catalyst	Conv. (%)	Benzene	Cyclohexanol	Cyclohexanone	Phenol
4-*O*-5 model compound	H-ZSM-5(60)	1.4	100	–	–	–
	CaO–H-ZSM-5(60)	2.3	5.3	–	–	94.7
	Ni–H-ZSM-5(60)	26.2	65.5	1.2	11.6	22.9
	Ni–CaO–H-ZSM-5(60)	47.3	10.1	20.6	3.2	66.1

Reactant	Catalyst	Conv. (%)	Toluene	Guaiacol		
α-*O*-4 model compound	–	2.7	30.3	69.7		
	H-ZSM-5(60)	3.6	5.8	29.5		
	CaO–H-ZSM-5(60)	7.5	48.7	51.3		
	Ni–H-ZSM-5(60)	99.5	46.5	53.5		
	Ni–CaO-H-ZSM-5(60)	100	49.4	50.5		

Reaction conditions: the amount of the reactant (β-*O*-4, 1-phenyl ethanol, 4-*O*-5 and α-*O*-4) was 2.5 mmol, m_reactant_:m_catalyst_ = 6:1, ethanol (30 mL), 140 °C, 1 MPa H_2_, 90 min, stirring at 700 rpm

Similarly, the conversion and product distributions of 4-*O*-5 and α-*O*-4 model compounds over different catalysts was also investigated (Table 2). It was found that the conversions of 4-*O*-5 and α-*O*-4 model compounds over H-ZSM-5(60) and CaO–H-ZSM-5(60) were extremely low. Other than this, the product distributions of 4-*O*-5 model compounds over Ni/CaO–H-ZSM-5(60) were also different from that over Ni/H-ZSM-5(60). There was no doubt that over Ni/CaO–H-ZSM-5(60), about 47% of the 4-*O*-5 model compounds had been converted mainly producing phenol and cyclohexanol with the selectivity of 66.1% and 20.6%, respectively. However, over Ni/H-ZSM-5(60), about 26% of 4-*O*-5 model compounds was converted, as well as benzene and phenol were deemed as the main products with the selectivity of 65.5% and 22.9%, respectively (Table 2). According to the results, the addition of CaO was beneficial to the conversion of 4-*O*-5 model compound. The C–O ether bond of 4-*O*-5 model compounds had different break sites over Ni/CaO–H-ZSM-5(60) and Ni/H-ZSM-5(60), resulting in a change in product distributions. The product distributions of α-*O*-4 conversion over these two catalysts [Ni/H-ZSM-5(60), Ni/CaO–H-ZSM-5(60)] was consistent (Table 2), the reason may be that the bond energy of the C–O ether bond in α-*O*-4 model compounds was lower, and it was easily cleaved during the hydrogenation of the metal Ni. It was well known that the acidity of H-ZSM-5 was beneficial to the hydrodeoxygenation reaction of biomass [40, 41]. However, it could be seen that Ni/H-ZSM-5 had quite low ability on breaking ether bond. The introduction of CaO significantly enhanced the cleavage of ether bonds, which would more favorable for lignin degradation to small molecular weight compounds. Therefore, we selected Ni/CaO–H-ZSM-5(60) and focused on the catalytic conversion of three typical lignin model compounds over it.

To ascertain the catalytic activity of Ni/CaO–H-ZSM-5(60), a series of additional experiments were performed about the effect of reaction temperature on β-*O*-4 conversion and the results were summarized in Table 3. The conversion activity of the β-*O*-4 was the lowest at 100 °C, then, along with the increasing of temperature, the conversion rate increased sharply. Note that the temperature maintained at 140–160 °C and the catalytic activity remained high. When the temperature was 140 °C, the catalytic activity was highest and β-*O*-4 conversion rate up to 100%, producing plenty of small aromatic monomers such as ethylbenzene, 1-phenylethanol, and guaiacol with the selectivity of 20.3%, 30.9% and 47.2%, respectively.

**Table 3:** The main product distributions of β-*O*-4 conversion at different temperature over Ni/CaO–H-ZSM-5(60)

Temperature (°C)	Conversion (%)	Selectivity (%)		
		Ethylbenzene	1-Phenylethanol	Guaiacol
100	16.7	2.8	46.4	49.5
120	77.2	4.0	45.7	49.1
140	100.0	20.3	30.9	47.2
160	93.2	11.0	37.1	50.8
180	66.6	3.3	44.1	52.0
200	46.0	2.8	36.2	61.0
250	65.7	5.6	29.2	64.7

Reaction conditions: β-*O*-4 (0.61 g, 2.5 mmol), Ni/CaO–H-ZSM-5(60) (0.102 g), ethanol (30 mL), 140 °C, 1 MPa H_2_, 60 min, stirring at 700 rpm

### Hydrogenolysis of β-*O*-4 model compound

**Fig. 7:**
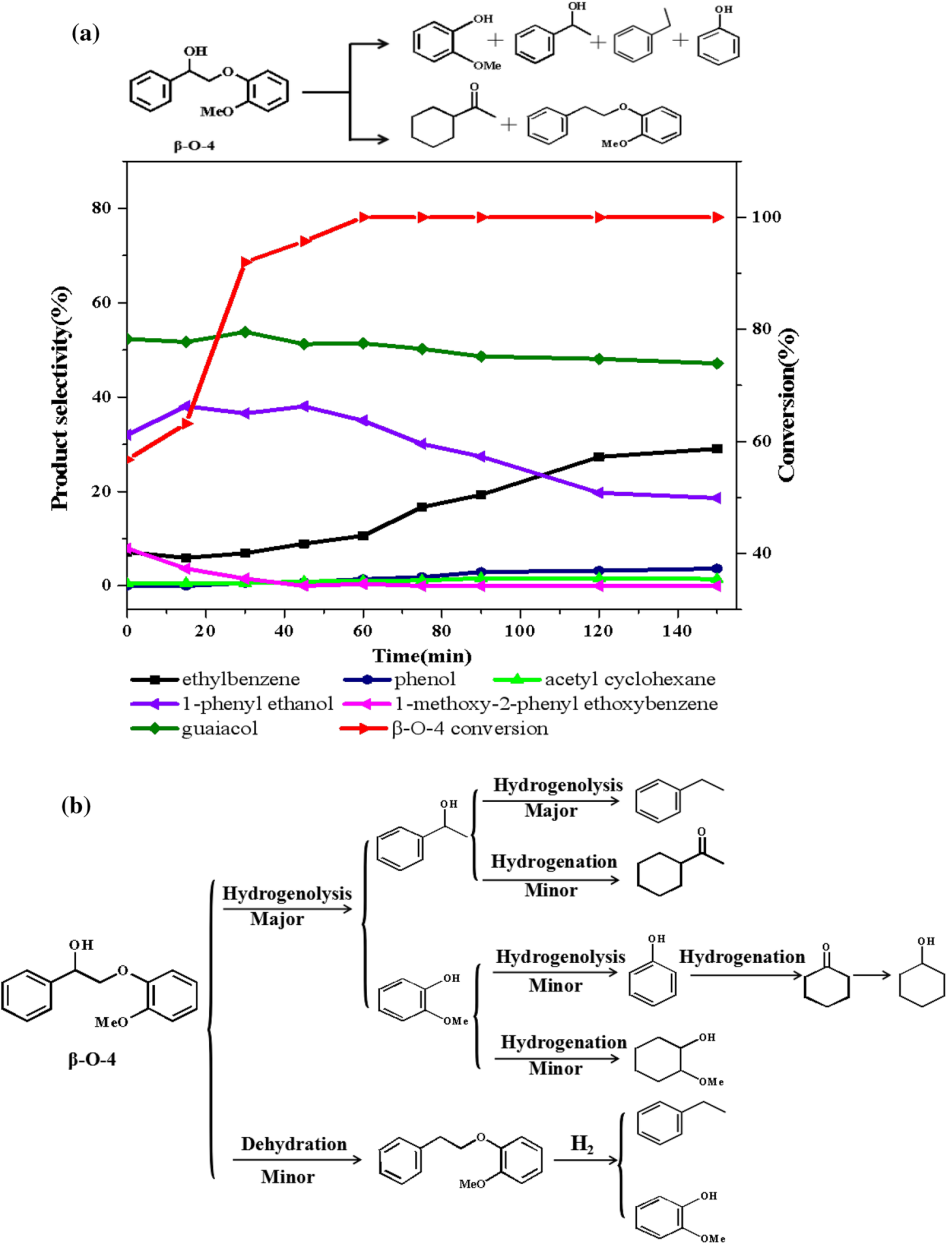
a The product distributions for the conversion of 2-(2-methoxyphenoxy)-1-phenylethanol (β-*O*-4) over Ni/CaO–H-ZSM-5(60) as a function of time. Reaction conditions: 2-(2-methoxyphenoxy)-1-phenylethanol (β-*O*-4) (0.61 g, 2.5 mmol), Ni/CaO–H-ZSM-5(60) (0.102 g), ethanol (30 mL), 140 °C, 1 MPa H_2_. **b** Two different reaction pathways of 2-(2-methoxyphenoxy)-1-phenylethanol (β-*O*-4) conversion over Ni/CaO-H-ZSM-5(60)

Figure 7a shown the product distributions for 2-(2-methoxyphenoxy)-1-phenylethanol (β-*O*-4) conversion as a function of time at 140 °C and 1 MPa H_2_. Over Ni/CaO–H-ZSM-5(60), about 92% of the β-*O*-4 had been converted in 30 min, producing 35.2% selectivity of 1-phenylethanol, 55.6% selectivity of guaiacol and 6.7% selectivity of ethylbenzene. During the reaction, only a small amount of dehydration product (1-methoxy-2-phenylethoxybenzene) was obtained. These results indicated that Ni/CaO–H-ZSM-5(60) catalyst had a high selectivity for breaking the β-*O*-4 linkage. As the reaction proceeded, the selectivity of guaiacol remained stable, whereas the selectivity of 1-phenylethanol decreased after reaching a maximum of 37.1% at 45 min, followed hydrogenolysis producing ethylbenzene.

**Fig. 8:**
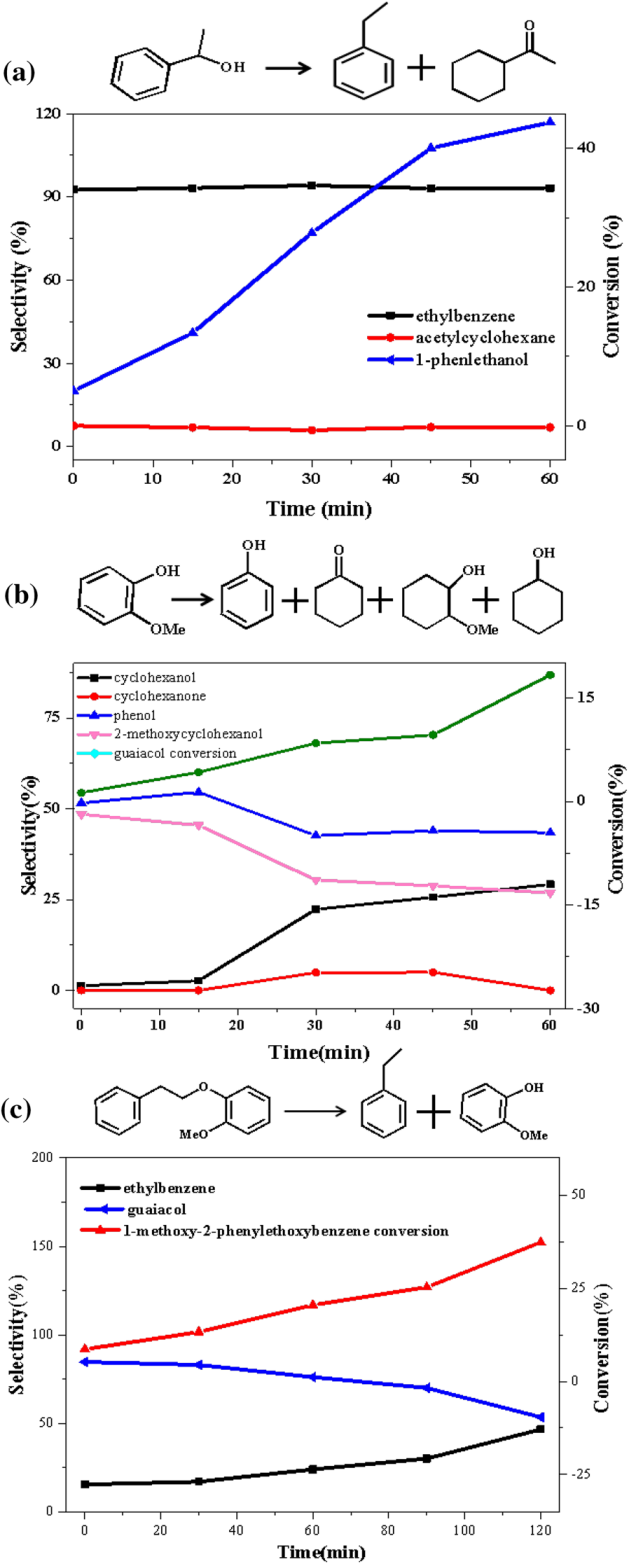
The product distributions for the conversions of **a** 1-phenylethanol, **b** guaiacol, and **c** 1-methoxy-2-phenylethoxybenzene over Ni/CaO-H-ZSM-5(60) as a function of time. Reaction conditions: reactant (2.5 mmol), Ni/CaO-H-ZSM-5(60) (0.102 g), ethanol (30 mL), 140 °C, 1 MPa H_2_, stirring at 700 rpm

In order to analysis the possible pathways for the C–O cleavage in 2-(2-methoxyphenoxy)-1-phenylethanol (β-*O*-4), a series of additional experiments as a function of time were performed. Figure 8 summarized the product distributions of 1-phenylethanol, guaiacol, and 1-methoxy-2-phenylethoxybenzene conversion in the presence of 1 MPa H_2_ at 140 °C. There was no doubt that over Ni/CaO–H-ZSM-5(60), about 48% of the 1-phenylethanol had been converted via two parallel reactions of hydrogenolysis and hydrogenation in 60 min, producing 93.1% selectivity of ethylbenzene and 6.9% selectivity of acetyl acetylcyclohexane (Fig. 8a). As the reaction proceeded, the ethylbenzene selectivity had remained around thirteen times that of acetylcyclohexane, indicating that 1-phenylethanol preferentially underwent hydrogenolysis over the Ni/CaO–H-ZSM-5(60) during the reaction. By a sharp contrast, guaiacol had a quite low conversion about 18% in 60 min under the same condition, producing cyclohexanol, phenol, and 2-methoxycyclohexanol with the selectivity of 29.2%, 43.4% and 26.9%, respectively. It was known that guaiacol contained functional groups of lignin, such as hydroxyl and methoxy groups. The bond energy of C–O in methoxy group is 247 kJ/mol, which is the weakest in guaiacol, while the bond energies of C–O in phenolic hydroxyl group so high that difficult to break (414 kJ/mol) [42]. Therefore, even under the condition of sufficient hydrogen, the oxygen in guaiacol also could not be completely removed and often results in phenol as the main catalytic product, which was corresponding with the result of Fig. 8b. In addition, the conversion of guaiacol mainly proceeded two parallel competitive pathways over Ni/CaO–H-ZSM-5(60) catalyst at 140 °C, at presence of 1 MPa H_2_. The first route was hydrogenation benzene ring of guaiacol, producing 2-methoxycyclohexanol as the major products. Another route was demethoxylation to form phenol, and further be converted to cyclohexanol and cyclohexanone via hydrogenation (Fig. 8b). Similarly, using 1-methoxy-2-phenylethoxybenzene as the reactant (shown in Fig. 8c), it could be seen that, over Ni/CaO–H-ZSM-5(60), about 37% of 1-methoxy-2-phenylethoxybenzene had been converted at 120 min, producing 46.5% selectivity of ethylbenzene and 53.5% selectivity of guaiacol. The result indicated that 1-methoxy-2-phenylethoxybenzene was an intermediate product of ethylbenzene and guaiacol, and if the reaction conditions permit, it would eventually be converted to ethylbenzene and guaiacol. At the same time, it also explained why 1-methoxy-2-phenylethoxybenzene appeared at the beginning of the reaction and eventually disappeared.

Combining with the results of Figs. 7a and 8, we deduced the corresponding reaction pathways about the conversion of 2-(2-methoxyphenoxy)-1-phenylethanol (β-*O*-4) over Ni/CaO–H-ZSM-5(60) which were presented in Fig. 7b. The conversion of β-*O*-4 mainly followed two parallel competitive pathways over Ni/CaO–H-ZSM-5(60) catalyst. The first one (major) was that the C–O bond of β-*O*-4 was selectively cleaved by initial hydrogenolysis to produce 1-phenylethanol and guaiacol. Since 1-phenylethanol over Ni/CaO–H-ZSM-5(60) catalyst was more easily converted than guaiacol (Fig. 8), the catalyst preferentially converts 1-phenylethanol to ethylbenzene (major) and acetylcyclohexane (minor) through two parallel reactions (hydrogenolysis and hydrogenation), whereas the selectivity of the products of guaiacol was less than 4%. Another pathway was that the formation of 1-methoxy-2-phenylethoxybenzene by dehydration of β-*O*-4, then, followed by hydrogenolysis to form ethylbenzene and guaiacol.

**Fig. 9:**
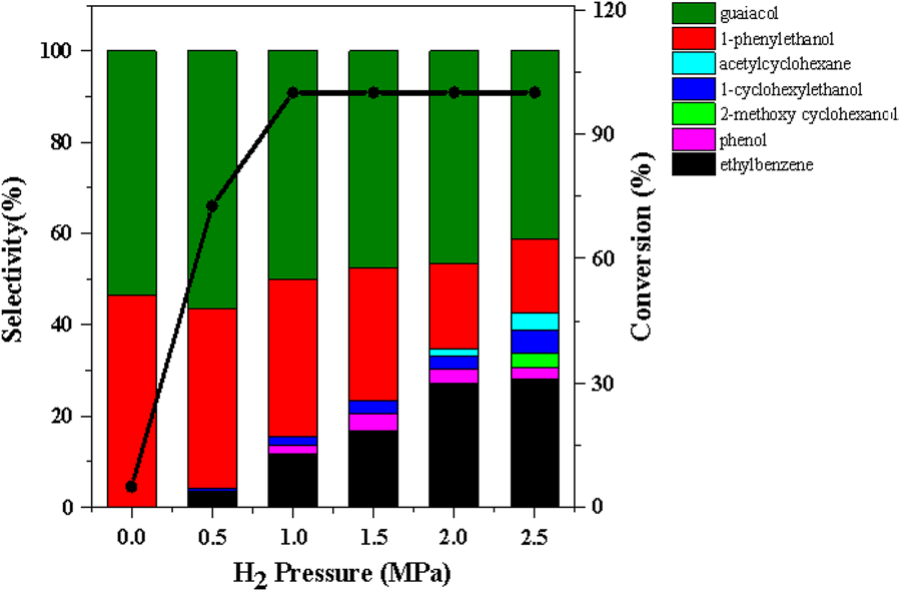
The product distributions for the conversion of 2-(2-methoxyphenoxy)-1-phenylethanol (β-*O*-4) over Ni/CaO–H-ZSM-5(60) under different H_2_ pressures. Reaction conditions: 2-(2-methoxyphenoxy)-1-phenylethanol (β-*O*-4) (0.61 g, 2.5 mmol), Ni/CaO–H-ZSM-5(60) (0.102 g), ethanol (30 mL), 140 °C, stirring at 700 rpm

Figure 9 clearly shown the product distributions of 2-(2-methoxyphenoxy)-1-phenylethanol (β-*O*-4) conversion varying H_2_ pressure. In the absence of H_2_, only 5% of 2-(2-methoxyphenoxy)-1-phenylethanol (β-*O*-4) was converted, indicating that H_2_ was indispensable for the hydrogenolysis pathway. As the H_2_ pressure increased the conversion of β-*O*-4 also increased. When the initial H_2_ pressure was 1 MPa, the conversion rate reached 100%, simultaneously, the main hydrogenolysis products of β-*O*-4 were 1-phenylethanol and guaiacol, with the highest selectivity of 34.5% and 50.1%, respectively. After that, the selectivity of guaiacol changed little, whereas the selectivity of 1-phenylethanol rapidly decreased after reaching a maximum at 1 MPa H_2_. The result indicated that 1-phenylethanol was more easily cleaved by hydrogenolysis than guaiacol, producing ethylbenzene and little amounts of acetylcyclohexane under conditions of sufficient hydrogen. Due to the stable properties of guaiacol, when the initial H_2_ pressure was 1 MPa, only a little part of the conversion to phenol. In addition, if the H_2_ pressure less than 2 MPa, no other hydrogenation product was found other than acetylcyclohexane. However, when the initial H_2_ pressure was 2.5 MPa, the hydrogenated product of guaiacol was found. Moreover, the selectivity of ethylbenzene, a hydrogenolysis product of 1-phenylethanol, did not change significantly at H_2_ pressures of 2 and 2.5 MPa. These results illustrated that low H_2_ pressure was favorable for the hydrogenolysis, while high H_2_ pressure not only favored the hydrogenation but also restrained the occurrence of hydrogenolysis.

### Hydrogenolysis of α-*O*-4 model compound

**Fig. 10:**
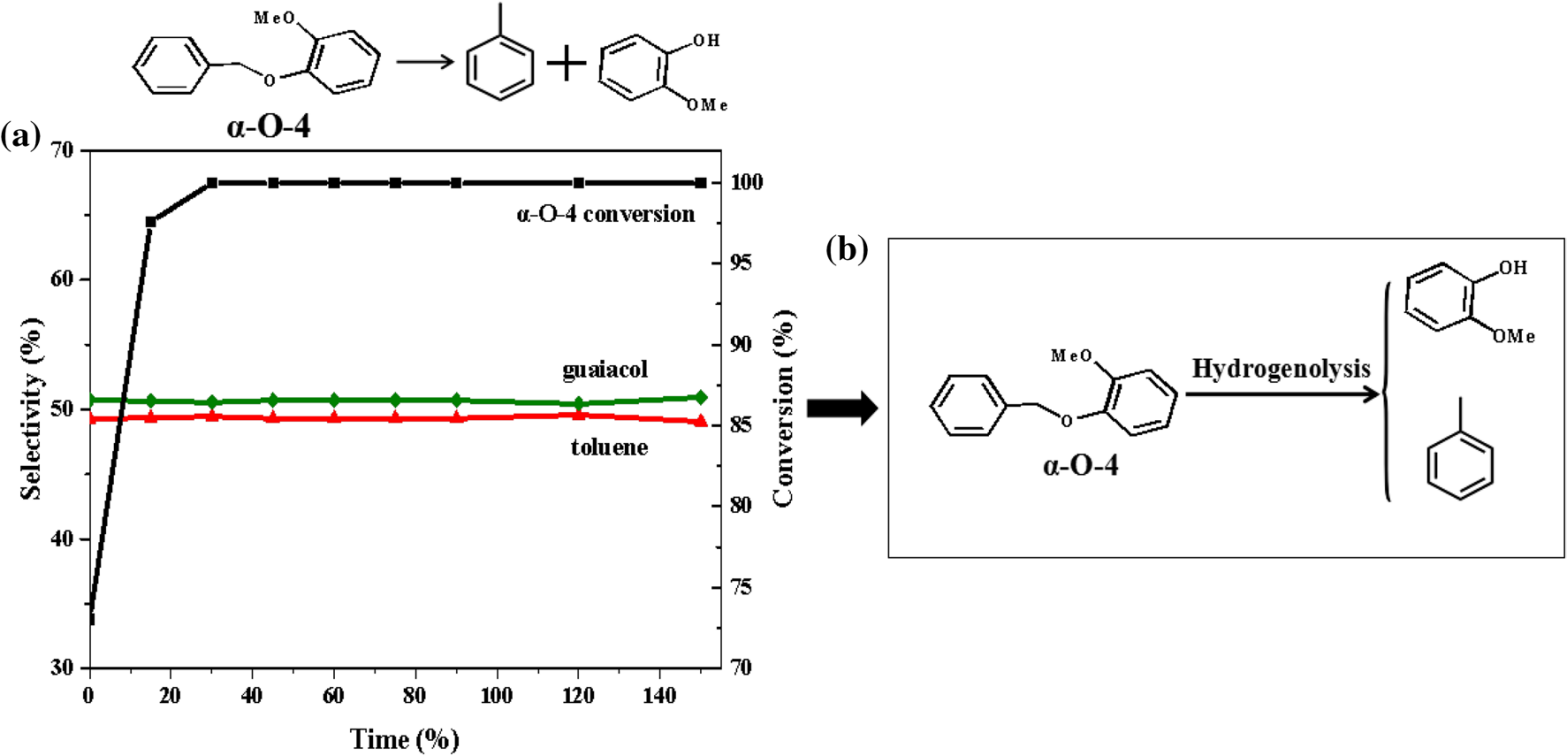
**a** The product distributions for the conversion of 2-methoxyphenyl anisole (α-*O*-4) over Ni/CaO–H-ZSM-5(60) as a function of time. Reaction conditions: 4-phenoxyphenol (2.5 mmol, 0.535 g), Ni/CaO–H-ZSM-5(60) (0.036 g), ethanol (30 mL), 140 °C, 1 MPa H_2_, stirring at 700 rpm. **b** Reaction pathway of 2-methoxyphenyl anisole (α-*O*-4) conversion over Ni/CaO–H-ZSM-5(60)

The reaction of 2-methoxyphenyl anisole (α-*O*-4) over Ni/CaO–H-ZSM-5(60) at 140 °C and 1 MPa H_2_ showed a very high conversion (Fig. 10). The C–O bond of 2-methoxyphenyl anisole (α-*O*-4) could be almost cleaved in 15 min (Fig. 10a), producing 49.4% selectivity of toluene and 50.6% selectivity of guaiacol. The result meant that the bond of α-*O*-4 was quite unstable, which was agree well with the report previously published [43].

During the reaction, toluene and guaiacol were resulted from hydrogenolysis of the C–O bond of 2-methoxyphenyl anisole (α-*O*-4). It indicated that a very low catalyst concentration could effectively break the C–O bond of 2-methoxyphenyl anisole (α-*O*-4) by hydrogenolysis, but no further conversion products of guaiacol were found. There were two reasons to explain this phenomenon. On the one hand, guaiacol was quietly stable and difficult to hydrogenate or hydrogenolysis, on the other hand, the catalyst with low concentration so that not sufficient for guaiacol fully converted. As above, the reaction pathway of the conversion of 2-methoxyphenyl anisole (α-*O*-4) was dominated through hydrogenolysis to generate toluene and guaiacol (Fig. 10b). After that, if the catalyst was sufficient, there would be a certain degree of hydrogenolysis of guaiacol to produce phenol (Fig. 8b).

**Fig. 11:**
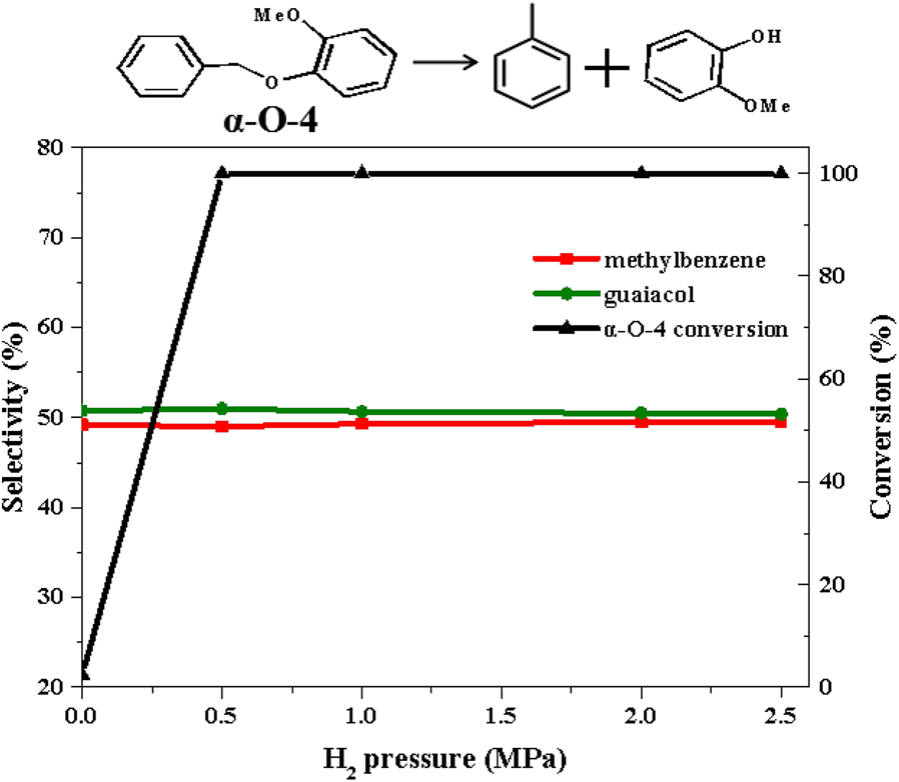
The product distributions for the conversion of 2-methoxyphenyl anisole (α-O-4) over Ni/CaO–H-ZSM-5(60) under different H_2_ pressures. Reaction conditions: 2-methoxyphenyl anisole (α-*O*-4) (0.535 g, 2.5 mmol), Ni/CaO–H-ZSM-5(60) (0.036 g), ethanol (30 mL), 140 °C, stirring at 700 rpm

Similarly, the product distributions of 2-methoxyphenyl anisole (α-*O*-4) conversion was recorded with varying H_2_ pressure (Fig. 11). It could be found that as the H_2_ pressure increased, the conversion of α-*O*-4 also increased. In the absence of H_2_, only 2.4% of 2-methoxyphenyl anisole (α-*O*-4) was converted. However, when the initial H_2_ pressure was 0.5 MPa, the conversion of α-*O*-4 reached 100%. It should be noted that the H_2_ pressure did not significantly influence the selectivity of toluene and guaiacol in the conversion products, showing that their chemical properties were stable and would not be converted under mild conditions.

### Hydrogenolysis of 4-*O*-5 model compound

**Fig. 12:**
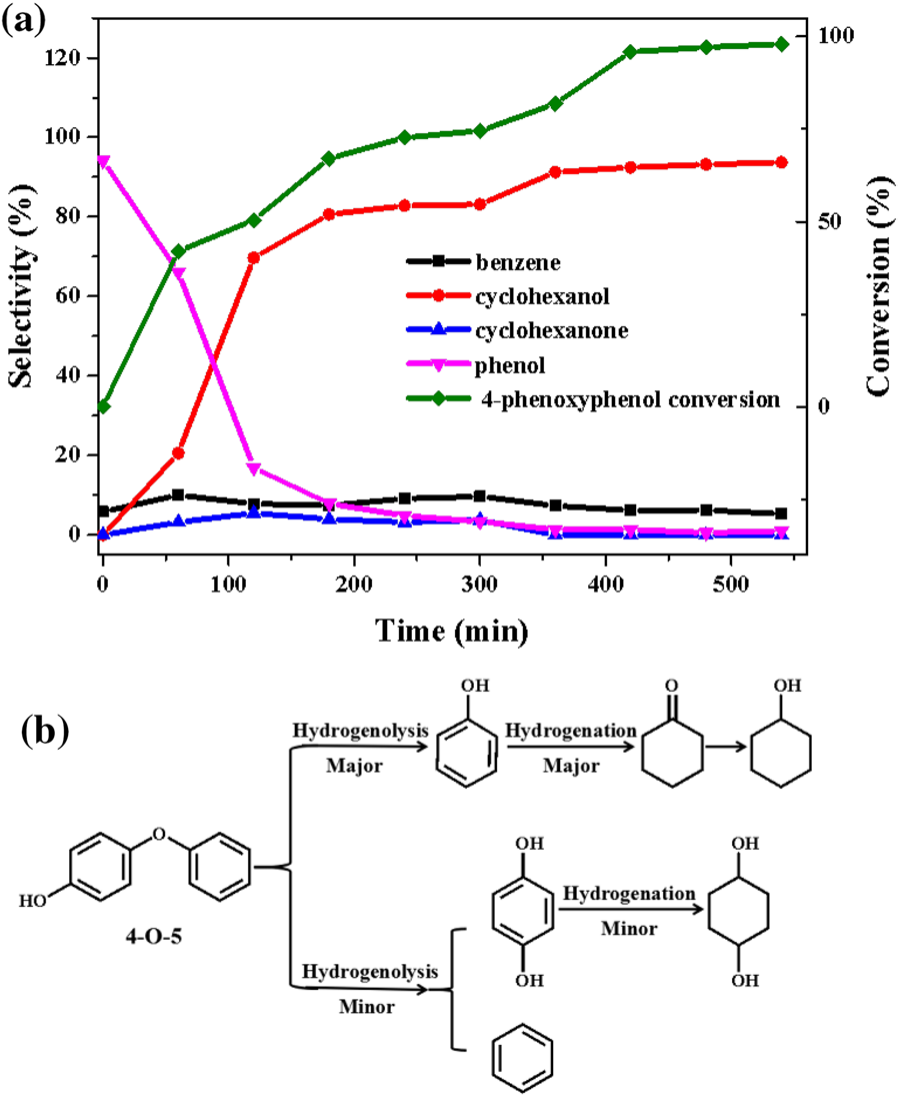
**a** The product distributions for the conversions of 4-phenoxyphenol (4-*O*-5) over Ni/CaO–H-ZSM-5(60) as a function of time. Reaction conditions: 4-phenoxyphenol (2.5 mmol, 0.465 g), Ni/CaO–H-ZSM-5(60) (0.0775 g), ethanol (30 mL), 140 °C, 1 MPa H_2_, stirring at 700 rpm. **b** Two different reaction pathways of 4-phenoxyphenol (4-*O*-5) conversion over Ni/CaO–H-ZSM-5(60)

As the third most abundant ether in lignin, 4-phenoxyphenol (4-*O*-5) had a very high ether bond energy compared to those in α-*O*-4 and β-*O*-4 model compounds. Therefore, the conversion of 4-*O*-5 model compound has been considered a challenge because of requiring the conditions of high temperature or supercritical ethanol to cleave the C–O bond. Figure 12a shown the product distributions for 4-phenoxyphenol conversion over Ni/CaO–H-ZSM-5(60) catalyst as a function of time at 140 °C and 1 MPa H_2_. About 40% of 4-*O*-5 had been converted in 60 min, producing 66.1%, 10.1%, 20.6% and 3.2% selectivity of phenol, benzene, cyclohexanol, and cyclohexanone, respectively. As the reaction proceeded, the selectivity of benzene remained stable, whereas the selectivity of phenol gradually decreased after reaching the maximum at 0 min, although phenol was considered to be the main product of 4-phenoxyphenol (4-*O*-5) via hydrogenolysis. The result meant that phenol could be rapidly converted to cyclohexanol over Ni/CaO–H-ZSM-5(60).

**Fig. 13:**
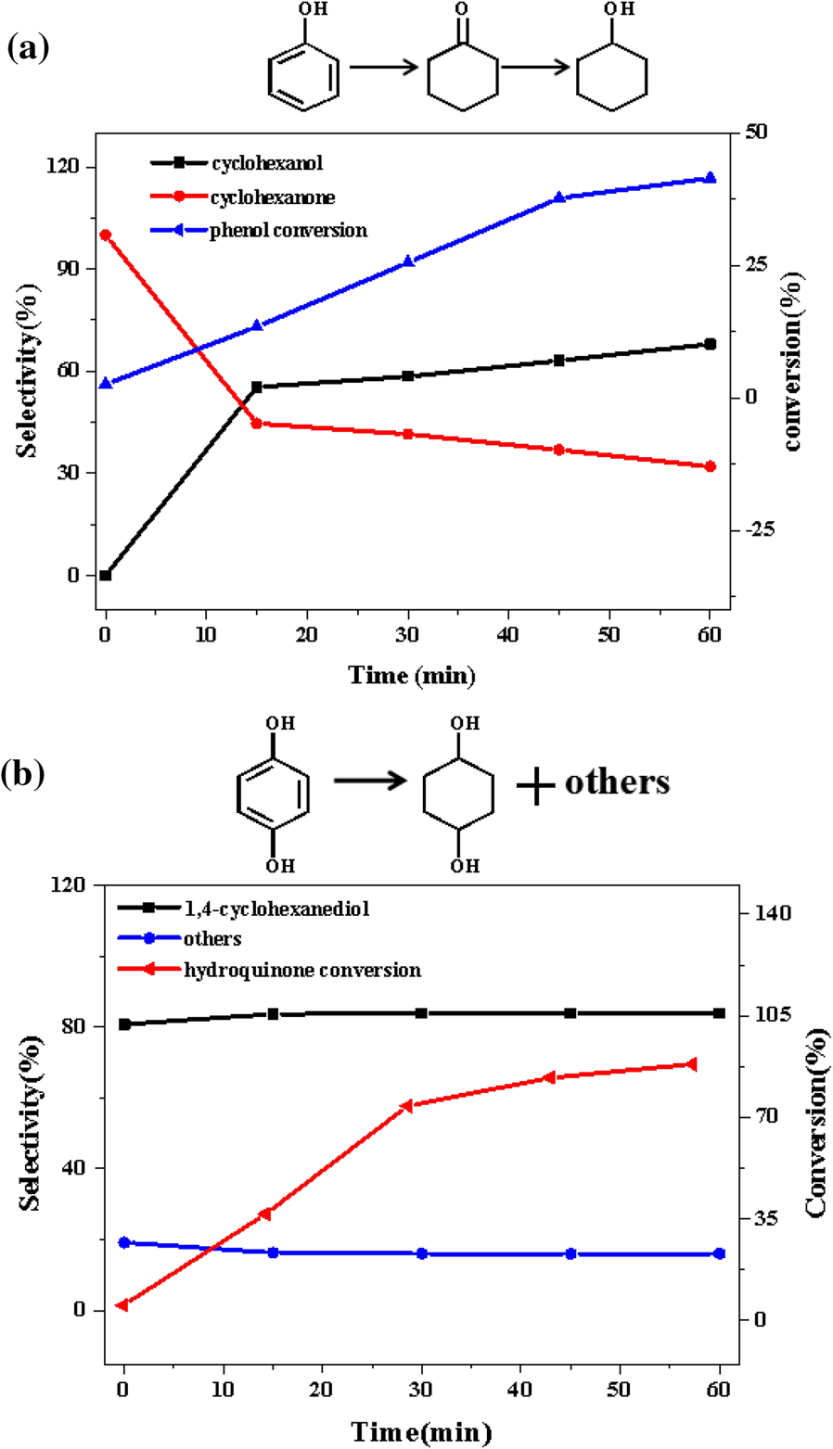
The product distributions for the conversion of **a** phenol and **b** hydroquinone over Ni/CaO–H-ZSM-5(60) as a function of time. Reaction conditions: phenol (2.5 mmol) or hydroquinone (2.5 mmol), Ni/CaO-H-ZSM-5(60) (0.0775 g), ethanol (30 mL), 140 °C, 1 MPa H_2_, stirring at 700 rpm

Compared with the pathways of breaking the ether C–O bond of β-*O*-4 and α-*O*-4, the chemistry for breaking the ether C–O bond in 4-phenoxyphenol (4-*O*-5) was more complex. Because phenol, benzene, and hydroquinone were supposed to be the main products after the initial hydrogenolysis of 4-phenoxyphenol, they were not a lot of existence in the product distributions of 4-phenoxyphenol. Therefore, it was speculated that phenol and hydroquinone might be intermediate products of a certain product. In order to explore the possible pathways of the conversion of 4-phenoxyphenol over Ni/CaO–H-ZSM-5(60), phenol and hydroquinone were used as reactants and the reaction conditions were consistent with those of 4-phenoxyphenol conversion (Fig. 13). Over Ni/CaO–H-ZSM-5(60), about 42% of the phenol had been converted via hydrogenation in 60 min, producing 68.0% selectivity of cyclohexanol and 32.0% selectivity of cyclohexanone (Fig. 13a). Among them, cyclohexanone was an intermediate product of hydrogenation of cyclohexanol, and would eventually be converted to cyclohexanol [44]. The conversion of hydroquinone versus reaction time was given in Fig. 13b, about 90% of hydroquinone was converted in 60 min, and the selectivity of the main product 1, 4-cyclohexanediol also reached more than 83.9%, indicating that hydroquinone could be easily converted than phenol (Fig. 13b). However, the selectivity of cyclohexanol in the 4-phenoxyphenol (4-*O*-5) conversion products was over 90%, but the selectivity of hydroquinone and 1,4-cyclohexanediol was extremely low that the quantitative analysis could not be performed (Fig. 12). Therefore, over Ni/CaO–H-ZSM-5(60), the C–O ether bond of 4-phenoxyphenol (4-*O*-5) was inclined to cleave producing phenol, and only a little amount of it could produce benzene and hydroquinone. As above, we deduced a corresponding reaction pathway about the conversion of 4-phenoxyphenol (4-*O*-5) over Ni/CaO–H-ZSM-5(60) which was presented in Fig. 12b. The conversion of 4-phenoxyphenol (4-*O*-5) mainly followed two parallel competitive pathways over Ni/CaO–H-ZSM-5(60) catalyst, the first reaction pathway (major) was that the C–O bond of 4-phenoxyphenol (4-*O*-5) was selectivity cleaved underwent the initial hydrogenolysis producing phenol, then followed by hydrogenation to form cyclohexanol. The second pathway (minor) was the hydrogenolysis reaction of 4-phenoxyphenol (4-*O*-5) producing benzene and hydroquinone.

### Recycling tests

**Fig. 14:**
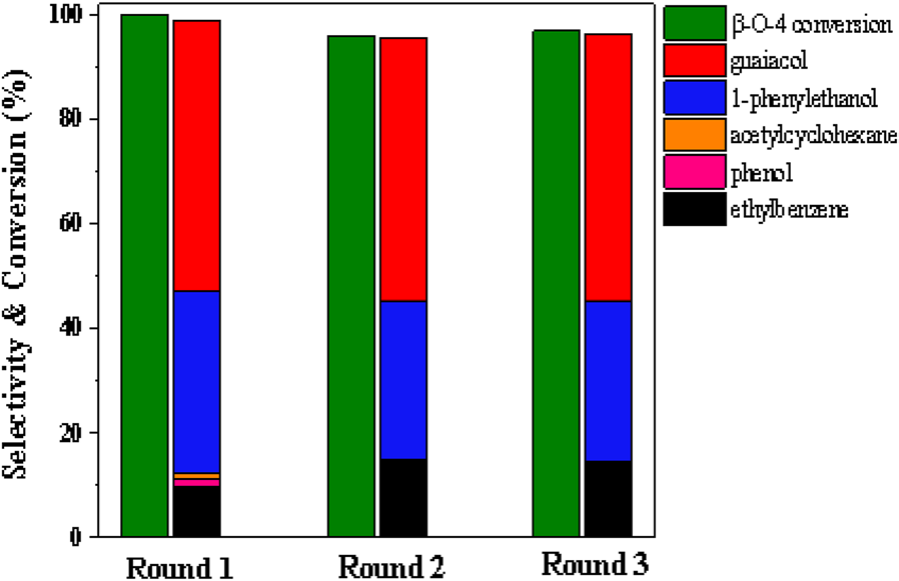
Recycling tests of Ni/CaO–H-ZSM-5(60) catalyst. Reaction conditions: β-*O*-4 (0.61 g, 2.5 mmol), Ni/CaO–H-ZSM-5(60) (0.102 g), ethanol (30 mL), 140 °C, 1 MPa H_2_, stirring at 700 rpm

The recycle ability of Ni/CaO–H-ZSM-5(60) was tested in 2-(2-methoxyphenoxy)-1-phenylethanol (β-*O*-4) reaction at 140 °C under 1 MPa. After the reaction, the used catalysts were subjected to an activation process with calcination and reduction. The initial recovery showed that, the Ni/CaO–H-ZSM-5(60) catalyst reacted at mild conditions (140 °C, 1 MPa H_2_) for 60 min and remained active after three cycles, and the conversion of reactant was above 98% in three runs (shown in Fig. 14). In addition, the selectivity of guaiacol was maintained at about 50% in three runs, and the selectivity of ethylbenzene increased to 14.3% after reaching 9.3% in the first reaction. Therefore, the selectivity of 1-phenylethanol was correspondingly reduced. Moreover, the selectivity of the other two by-products, acetylcyclohexane and phenol, continuously decreased until they disappear.

## Conclusion

The C–O ether bonds in the β-*O*-4, α-*O*-4 and 4-*O*-5 model compounds are typical and abundant in native lignin. In this study, these ether bonds were selectivity cleaved by a novel Ni/CaO–H-ZSM-5(60) catalyst to produce small molecular compounds in ethanol under very mild conditions (140 °C, 1 MPa H_2_). The C–O bond of α-*O*-4 was cleaved by direct hydrogenolysis. However, the C–O bonds of β-*O*-4 and 4-*O*-5 were cleaved via two parallel reactions, but the ways of breaking C–O bonds were different. Moreover, H_2_ was indispensable for the hydrogenolysis and hydrogenation, and low H_2_ pressure favored hydrogenolysis, while high H_2_ pressure favored hydrogenation.

## Additional files

**Additional file 1: Figure S1.** The H^1^NMR spectrums of three lignin model compound. (a) 2-(2-Methoxyphenoxy)-1-phenylethanol (β-O-4), (b) 2-methoxyphenyl anisole (α-O-4), and (c) 2-methoxy-1-phenylethoxybenzene.

**Additional file 2: Figure S2.** Specific preparation process of Ni/CaO-H-ZSM-5(60) catalyst.

**Additional file 3: Figure S3.** Specific reaction process of β-O-4 conversion over Ni/CaO-H-ZSM-5(60) catalyst and the main analytical approaches.

## Abbreviations

β-*O*-4: 2-(2-methoxyphenoxy)-1-phenylethanol
α-*O*-4: 2-methoxyphenylanisole
4-*O*-5: 4-phenoxyphenol
S: sinapyl alcohol
G: coniferyl alcohol
H: p-coumaryl alcohol
HTC: hydrotalcite
MTA: methanol to aromatic hydrocarbons
DP: deposition–precipitation
XRD: X-ray diffraction
TEM: transmission electron microscopy
BET: Brunauer–Emmett–Teller
BJH: Barrett–Joyner–Halenda
FT-IR: Fourier transform infrared spectroscopy
ICP-AES: inductively coupled plasma atomic emission spectrometry

## References

[1] Li C, Zhao X, Wang A, Huber GW, Zhang T (2015). Catalytic transformation of lignin for the production of chemicals and fuels. Chem Rev.

[2] Yang J, Lu X, Liu X, Xu J, Zhou Q, Zhang S (2017). Rapid and productive extraction of high purity cellulose material via selective depolymerization of the lignin–carbohydrate complex at mild conditions. Green Chem.

[3] Liao Y, Liu Q, Wang T, Long J, Zhang Q, Ma L, Liu Y, Li Y (2014). Promoting hydrolytic hydrogenation of cellulose to sugar alcohols by mixed ball milling of cellulose and solid acid catalyst. Energy Fuels.

[4] Noda Y, Wongsiriwan U, Song CP, Yeboah Y (2012). Sequential combination of acid and base for conversion of cellulose. Energy Fuels.

[5] Huber GW, Iborra S, Corma A (2006). Synthesis of transportation fuels from biomass: chemistry, catalysts, and engineering. Chem Rev.

[6] Evtuguin DV, Neto CP, Silva AMS, Domingues PM, Amado FML, Faix D, Robert O (2001). Comprehensive study on the chemical structure of dioxane lignin from plantation *Eucalyptus globulus* wood. J Agric Food Chem.

[7] Hu L, Pan H, Zhou Y, Zhang M (2011). Methods to improve lignin’s reactivity as a phenol substitute and as replacement for other phenolic compounds: a brief review. BioResources.

[8] Zakzeski J, Bruijnincx PCA, Jongerius AL, Weckhuysen BM (2010). The catalytic valorization of lignin for the production of renewable chemicals. Chem Rev.

[9] Wang H, Zhang L, Deng T, Ruan H, Hou X, Cort JR, Yang B (2016). ZnCl_2_ induced catalytic conversion of softwood lignin to aromatics and hydrocarbons. Green Chem.

[10] Kloekhorst A, Heeres HJ (2015). Catalytic hydrotreatment of alcell lignin using supported Ru, Pd, and Cu catalysts. ACS Sustain Chem.

[11] Hidajat MJ, Riaz A, Kim J (2018). A two-step approach for producing oxygen-free aromatics from lignin using formic acid as a hydrogen source. J Chem Eng.

[12] Ren X, Chen J, Li G, Wang Y, Lang X, Fan S (2018). Thermal oxidative degradation kinetics of agricultural residues using distributed activation energy model and global kinetic model. Bioresour Technol.

[13] De S, Zhang J, Luque R, Yan N (2016). Ni-based bimetallic heterogeneous catalysts for energy and environmental applications. Energy Environ Sci.

[14] Deepa AK, Dhepe PL (2015). Lignin depolymerization into aromatic monomers over solid acid catalysts. ACS Catal.

[15] Xia S, Guo X, Mao D, Shi Z, Wu G, Lu G (2014). Biodiesel synthesis over the CaO–ZrO_2_ solid base catalyst prepared by a urea–nitrate combustion method. RSC Adv.

[16] Pandey MP, Kim CS (2011). Lignin depolymerization and conversion: a review of thermochemical methods. Chem Eng Technol.

[17] Zakzeski J, Jongerius AL, Bruijnincx PCA, Weckhuysen BM (2012). Catalytic lignin valorization process for the production of aromatic chemicals and hydrogen. Chemsuschem.

[18] Konnerth H, Zhang J, Ma D, Prechtl MHG, Nan N (2015). Base promoted hydrogenolysis of lignin model compounds and organosolv lignin over metal catalysts in water. Chem Eng Sci.

[19] Bengoechea MO, Hertzberg A, Miletić N, Arias PL, Barth T (2015). Simultaneous catalytic de-polymerization and hydrodeoxygenation of lignin in water/formic acid media with Rh/Al_2_O_3_, Ru/Al_2_O_3_, and Pd/Al_2_O_3_, as bifunctional catalysts. J Anal Appl Pyrolysis.

[20] Long J, Zhang Q, Wang T, Zhang X, Xu Y, Ma L (2014). An efficient and economical process for lignin depolymerization in biomass-derived solvent tetrahydrofuran. Bioresour Technol.

[21] Sutradhar N, Sinhamahapatra A, Pahari SK, Pal P, Bajaj HC, Mukhopadhyay I, Panda AB (2011). Controlled synthesis of different morphologies of MgO and their use as solid base catalysts. J Phys Chem C.

[22] Sturgeon MR, O’Brien MH, Ciesielski PN, Katahira R, Kruger JS, Chmely SC, Hamlin J, Lawrence K, Hunsinger GB, Foust TD, Baldwin RM, Biddy MJ, Beckham GT (2014). Lignin depolymerisation by nickel supported layered-double hydroxide catalysts. Green Chem.

[23] Krugser JS, Cleveland NS, Zhang S, Katahira R, Black BA, Chupka GM, Lammens T, Hamilton PG, Biddy MJ, Beckham GT (2016). Lignin depolymerization with nitrate-intercalated hydrotalcite catalysts. ACS Catal.

[24] Song Q, Wang F, Cai J, Wang Y, Zhang J, Yu W, Xu J (2013). Lignin depolymerization (LDP) in alcohol over nickel-based catalysts via a fragmentation–hydrogenolysis process. Energy Environ Sci.

[25] Patil PT, Armbruster U, Richter M, Martin A (2011). Heterogeneously catalyzed hydroprocessing of organosolv lignin in sub- and supercritical solvents. Energy Fuels.

[26] Runnebaum RC, Nimmanwudipong T, Limbo RR, Block DE, Gates BC (2012). Conversion of 4-methylanisole catalyzed by Pt/γ-AlO and by Pt/SiO-AlO: reaction networks and evidence of oxygen removal. Catal Lett.

[27] Kassaye S, Pagar C, Pant KK, Jain S, Gupta R (2016). Depolymerization of microcrystalline cellulose to value added chemicals using sulfate ion promoted zirconia catalyst. Bioresour Technol.

[28] Zhang S, Yang M, Shao J, Yang H, Zeng K, Chen Y, Luo J, Agblevor FA, Chen H (2018). The conversion of biomass to light olefins on Fe-modified ZSM-5 catalyst: effect of pyrolysis parameters. Sci Total Environ.

[29] Niu X, Gao J, Wang K, Miao Q, Dong M, Wang G, Qin WZ, Wang J (2017). Influence of crystal size on the catalytic performance of H-ZSM-5 and Zn/H-ZSM-5 in the conversion of methanol to aromatics. Fuel Process Technol.

[30] Zhang D, Zhao YP, Fan X, Liu ZQ, Wang RY, Wei XY (2018). Catalytic hydrogenation of levulinic acid into gamma-valerolactone over Ni/HZSM-5 catalysts. Catal Surv Asia.

[31] Melligan F, Mhb H, Kwapinski W, Leahy JJ (2012). Hydro-pyrolysis of biomass and online catalytic vapor upgrading with Ni-ZSM-5 and Ni-MCM-41. Energy Fuels.

[32] Rahimi A, Azarpira A, Kim H, Ralph J, Stahl SS (2013). Chemoselective metal-free aerobic alcohol oxidation in lignin. J Am Chem Soc.

[33] Jin X, Shen J, Yan W, Zhao M, Thapa PS, Subramaniam B, Chaudhari RV (2015). Sorbitol hydrogenolysis over hybrid Cu/CaO–Al_2_O_3_ catalysts: tunable activity and selectivity with solid base incorporation. ACS Catal.

[34] Maia AJ, Oliveira BG, Esteves PM, Louis B, Lam YL, Pereira MM (2011). Isobutane and *n*-butane cracking on Ni-ZSM-5 catalyst: effect on light olefin formation. Appl Catal A.

[35] Vitale G, Molero H, Hernandez E, Aquino S, Birss V, Pereira-Almao P (2013). One-pot preparation and characterization of bifunctional Ni-containing ZSM-5 catalysts. Appl Catal A.

[36] Li H, Dong L, Zhao L, Cao L, Gao J, Xu C (2017). Enhanced adsorption desulfurization performance over mesoporous ZSM-5 by alkali treatment. Ind Eng Chem Res.

[37] Xu C, Tang SF, Sun X, Sun Y, Li G, Qi J, Li X, Li X (2017). Investigation on the cleavage of β-*O*-4 linkage in dimeric lignin model compound over nickel catalysts supported on ZnO–Al_2_O_3_ composite oxides with varying Zn/Al ratios. Catal Today.

[38] Kristianto I, Limarta SO, Lee H, Ha JM, Suh DJ, Jae J (2017). Effective depolymerization of concentrated acid hydrolysis lignin using a carbon-supported ruthenium catalyst in ethanol/formic acid media. Bioresour Technol.

[39] Rahimi A, Ulbrich A, Coon JJ, Stahl SS (2014). Formic-acid-induced depolymerization of oxidized lignin to aromatics. Nature.

[40] Kalogiannis KG, Stefanidis SD, Karakoulia SA, Triantafyllidisb KS, Yiannoulakisc H, Mihailofa C, Lappasa AA (2018). First pilot scale study of basic vs acidic catalysts in biomass pyrolysis: deoxygenation mechanisms and catalyst deactivation. Appl Catal B Environ.

[41] Navarro RM, Guil-Lopez R, Fierro JLG, Motaa N, Jiménezb S, Pizarrob P, Coronadob JM, Serranob DP (2018). Catalytic fast pyrolysis of biomass over Mg-Al mixed oxides derived from hydrotalcite-like precursors: influence of Mg/Al ratio. J Anal Appl Pyrolysis.

[42] Yang L, Seshan K, Li Y (2016). A review on thermal chemical reactions of lignin model compounds. Catal Today.

[43] He J, Zhao C, Lercher JA (2012). Ni-catalyzed cleavage of aryl ethers in the aqueous phase. J Am Chem Soc.

[44] He J, Zhao C, Lercher JA (2014). Impact of solvent for individual steps of phenol hydrodeoxygenation with Pd/C and HZSM-5 as catalysts. J Catal.

