# Supplementary figures and images for: Highly selective cleavage C–O ether bond of lignin model compounds over Ni/CaO–H-ZSM-5 in ethanol
Source: BMC Chem. 2019 Mar 26;13(1):36. doi: 10.1186/s13065-019-0557-z (PMC6661968; doi:10.1186/s13065-019-0557-z)

**Additional file 1.**

**(a)**

**(b)**


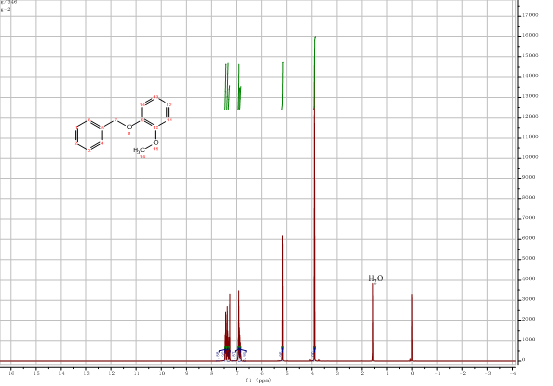


**(c)**

Supplement: Supplementary file 1 — Additional file 1: Figure S1. The H1NMR spectrums of three lignin model compound. (a) 2-(2-Methoxyphenoxy)-1-phenylethanol (β-O-4), (b) 2-methoxyphenyl anisole (α-O-4), and (c) 2-methoxy-1-phenylethoxybenzene. [file 13065_2019_557_MOESM1_ESM.doc]

**Additional file 2.**


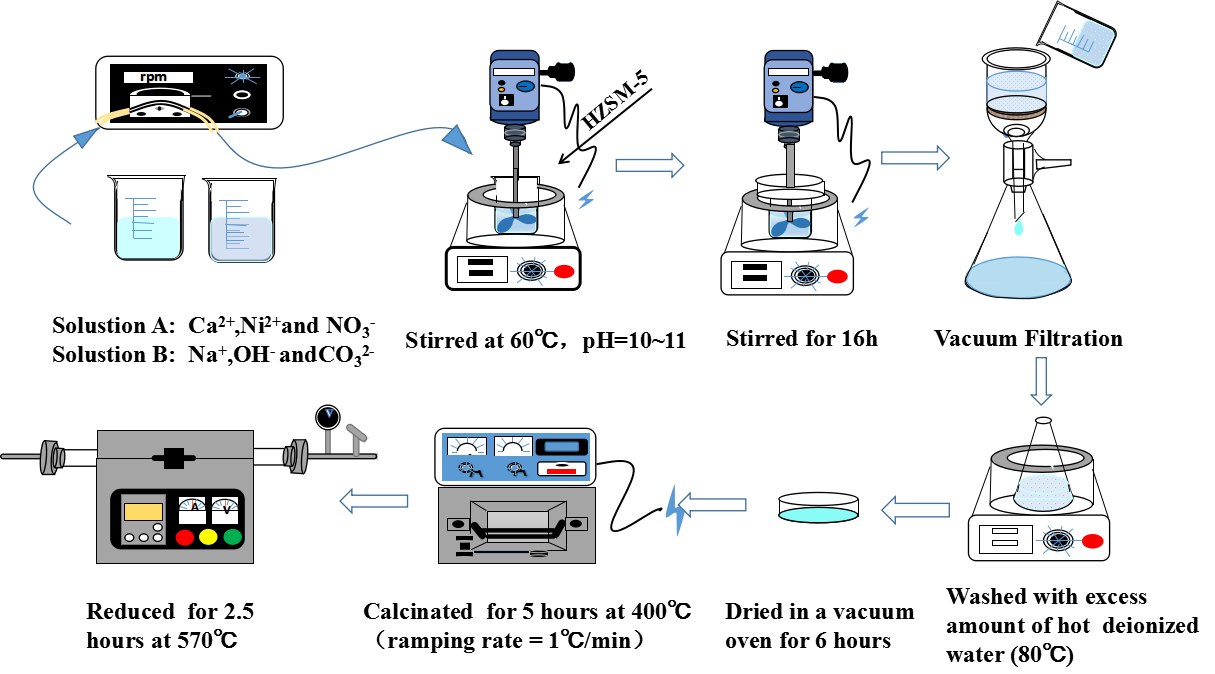

Supplement: Supplementary file 2 — Additional file 2: Figure S2. Specific preparation process of Ni/CaO-H-ZSM-5(60) catalyst. [file 13065_2019_557_MOESM2_ESM.doc]

**Additional file 3.**


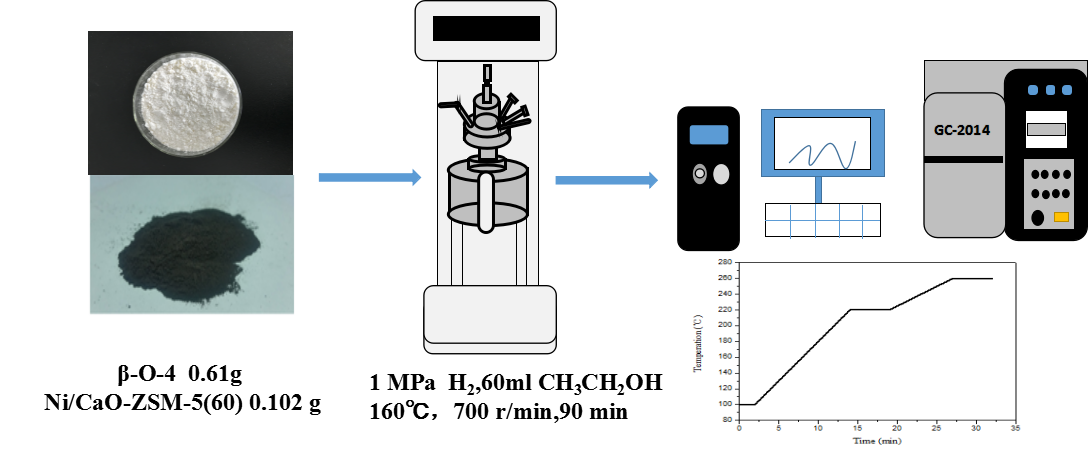

Supplement: Supplementary file 3 — Additional file 3: Figure S3. Specific reaction process of β-O-4 conversion over Ni/CaO-H-ZSM-5(60) catalyst and the main analytical approaches. [file 13065_2019_557_MOESM3_ESM.doc]
